# A Sanitation Argument for Clean Indoor Air: Meeting a Requisite for Safe Public Spaces

**DOI:** 10.3389/fpubh.2022.805780

**Published:** 2022-02-09

**Authors:** Anthony Joseph Leonardi, Asit Kumar Mishra

**Affiliations:** ^1^Bloomberg School of Public Health, Johns Hopkins University, Baltimore, MD, United States; ^2^MaREI Centre, Ryan Institute & School of Engineering, College of Science and Engineering, National University of Ireland Galway, Galway, Ireland

**Keywords:** airborne, SARS—CoV–2, aerosol, HVAC, HEPA, sanitation

## Introduction

In public health terms, “sanitation” refers to a public health implementation of hygienic standards and practices meant to address transmissible diseases like Malaria and Cholera in industrial and public settings like factories, schools, and resorts (1). We propose the management of air given the current pandemic with an airborne pathogen (2). Sanitation has had a stable history as a primary focus in the field of public health engineering, responsible for potable water, waste management, and control of mosquito breeding-grounds (1, 3). Since addressed by a sanitation approach, the effective handling of vector media has made outbreaks and epidemics like the cholera outbreak of 1,911 in New York City unrepeated in the USA (1). However, rarely have pathogens been met with mitigations and public health sanitation measures considering airborne transmission, save for sanitariums and open-air schools for Tuberculosis and the “Fresh Air” movement during the 1918 Influenza Pandemic, which were both caused by pathogens spreading by aerosols (4–6). In such a rare, but notable example in 1918, an open-air hospital in Boston was retrospectively found to benefit the staff by reducing Influenza infection (7). Given our current pandemic, we believe such ventilation measures should be readopted and the air should be sanitized.

As new evidence shows airborne pathogens such as SARS-Cov-2 spread *via* aerosols, we should refine what is a nebulous attribution of responsibility in mitigating the spread of airborne pathogens indoors and assign it under the purview of public health sanitation and engineering in order to effectively manage indoor air (2). A building's ventilation system is critical to maintaining a healthy work environment (8). Humans breathe in many times more air than our food or water intake—around 6 liters/minute (7). Therefore, we argue for the sanitation of air under the domain of public health environmental engineering, and echo the calls for a necessary paradigm shift *via* measures such as ventilation and filtration (8).

## Discussion

### Generation of Respiratory Aerosols

Many viruses including SARS-CoV-2 have ample evidence of primarily airborne transmission (8).

Generation of respiratory aerosols is not limited to aerosols generating medical procedures and is observed for many day-to-day activities like breathing, talking, shouting, coughing and sneezing, and singing (9–12). Emissions increase with airflow velocity and speech volume (13, 14). The expelled aerosols have a multimodal size distribution: 0.1, 0.2–0.8, 1.5–1.8, and 3.5–5.0 microns, while coughing and talking also have modes at 123 microns and 145 microns, respectively, though the large particles during both talking and coughing are still under 5 microns in size (2). Smaller sized aerosols are generated deeper in the respiratory tract (2).

Most exhaled aerosols are under 5 microns (15, 16). Normal breathing produces hundreds to thousands of such particles per liter of exhaled air (16–18). Due to such small size, these particles can be respired (19). For every particle over 100 microns produced during speech, 100 to 1,000 particles under 100 microns in size (10). Blustery expulsions, like a sneeze or a cough, can produce numerous aerosols in a short period, but talking and breathing are continuous action and a cause for greater concern (20), especially when an infectious person does not display symptoms. A minute of loud conversation can produce thousands of droplets every second, of which, about a thousand particles could contain virus and these can remain afloat for 8 mins or more (21).

While a historical 5 micron boundary has cropped up to distinguish between aerosols and droplets, a 100 micron boundary is supported by evidence (2, 22, 23). Stokes law for small particles subject to laminar flow can provide a simple approximation of their terminal velocity, thus providing an idea of how long they may stay afloat:


(1) $$\begin{array}{l} u_{p}\;=\;\frac{g{\rho_{p}d}_{p}^{2}C}{18\eta } \end{array}$$

where “g” is the acceleration due to gravity, “η” is the dynamic viscosity of air, “ρ_p_” is the density of the particle, “d_p_” is the diameter of the particle, and C is the Cunnigham slip correction factor (to account for slippage, leading to reduced air resistance, relevant when particle size becomes of the order of the mean free path of air molecules) (24). In still air, a 100 micron particle released at a 1.5 m height can stay in the air for ~5 s, while traversing ~2 m. Similarly, a 10 micron particle can stay suspended for ~17 min, a 5 micron particle for ~33 min and a 1 micron particle for over 12 h (2, 17, 25). A one micron respiratory aerosols is about a thousand times larger than a single virion. It can contain enough of the virus and stay afloat for hours. Studies have found smaller aerosols to be enriched with infectious pathogens (15, 19, 26, 27). Since room air is rarely still, these particles can get further, especially while aided by violent exhalation events like sneezing or coughing (28). Modeling shows that large droplets over 100 micron are only likely to be the dominant mode of infection within 0.2 m (talking) or 0.5 m (coughing) of an infectious person (29). This makes sense when you consider that the concentration of exhaled aerosols is highest closest to the source, in this case, the infectious person. Risks of infection from aerosols will be quite high close to the source (2), highest when the infected and the exposed individuals are positioned so close that breathing flows can approach each other's faces, with complex flow interactions, difficult to predict (20).

### Summary of Evidence for Airborne Spread of COVID-19

Greenhalgh et al. (30), succinctly summarized the evidence that strongly indicates COVID-19 is airborne. The following are some key points from their work.

- Long-range transmission of the disease and overdispersion of the basic reproduction number (R0). These are consistent with airborne transmission but cannot be adequately explained depending on droplets and fomites (31).
- Transmission between people who were never in each other's physical presence, as evidenced from outbreaks in quarantine hotels (32).
- Asymptomatic or presymptomatic transmission, where the infectious person is not sneezing or coughing, accounts for 33 to 59% of transmissions worldwide, indicating mostly airborne transmission and not droplets (33).
- The disease transmits much more easily indoors than outdoors (34), and transmission can be mitigated by good indoor ventilation (35–38).
- Despite strict contract and droplet precautions and use of relevant personal protective equipment (PPE) (against droplets only), nosocomial infections have unfortunately occurred (39).
- Viable SARS-CoV-2 has been detected in the air in laboratory studies (40, 41) as well as in spaces with infected occupants, without any so-called aerosol generating medical procedures being undertaken (42, 43). Exhalation of infectious aerosols have now been documented in both animal models (44) and in humans (26).
- SARS-CoV-2 has been traced to locations in buildings that could only be reached *via* aerosols, like air filters in air handling units of hospitals and the air conditioning vents/ducting (45)
- Animal models where transmission of SARS-CoV-2 occurred between animals whose cages were connected by a ducting network that can only be negotiated by aerosols and not droplets (46). It has also been shown in animal models that placing surgical masks around cages of infectious individuals reduced transmission (47). Animal models also show the aerosol exposure more likely leading to more severe disease and efficient transmission (48, 49).

Several in-depth *post-hoc* analysis of outbreaks have shown that transmission was most likely through aerosols, as opposed to droplets or fomites, like, a department store in China (50), a party traveling in buses (51), the Skagit Valley Chorale (52), and the outbreak on the Diamond Princess cruise ship (53).

### Mitigation

Relative humidity of indoor air impacts the equilibrium size of exhaled aerosols particles (and thus how long they are suspended in air and the distance they can traverse), the viability of viruses in the particles, and our immune defenses (mucociliary clearance) (2). A relative humidity of 40–60% indoors could reduce possibilities of transmission (54, 55).

Both the volume of ventilation and air flow patterns in an occupied space have an impact on airborne transmission of viruses (2, 20). Good ventilation can improve indoor air quality and benefit health, comfort, and office work performance, while also reducing occurrences of allergic and asthmatic incidents (56, 57). It is important to assure that, like food or waterborne diseases, we can reduce risks of airborne diseases through appropriate engineering measures (8).

Standards recommend minimum ventilation rates for buildings based on either needs for maintaining acceptable indoor air quality (58) or needs for infection prevention (59). The ventilation in a specific building depends on the intended use of the space, like a school, vs. office buildings, vs. residences, vs. hospital wards, due to differences in occupancy density, layouts, hours of occupancy, and infection prevention needs. Type of ventilation system also affects the chances of infection transmission. In an ideal world, when we can be sure of who is infectious, personalized extraction ventilation for infectious persons can dramatically reduce infection transmission risks (20). However, when a virus can be transmitted by persons exhibiting no symptoms, we would have to provide personalized ventilation and personalized extraction to every occupant, which can quickly become prohibitively costly. An increase in ventilation volume need not always correspond with a reduction in risks (60), implying ventilation volume alone should not be used as an indicator for ventilation performance in actual buildings (20).

Improved ventilation has also been related to reduction in SBS (sick building syndrome) symptoms and relative risks of respiratory illness (61), particularly for the elderly (62), improved comfort and lowering sick absence (schools and offices) (63), and improved productivity (even offsetting any additional energy costs) (64, 65). Models of infectious disease transmission show that improved ventilation can mitigate outbreaks of influenza (66), seasonal variations in ventilation (less ventilation during winter) can increase risks of airborne disease transmission in classrooms (67), improved air quality reduces transmission risks of several airborne pathogens in clinics (68), and can also reduce disease transmission risks at a city level (69). A disease that is airborne and has epidemic proportions around the world, is tuberculosis and there are several studies linking improvement in ventilation with reduction in risks of tuberculosis infection (70, 71).

Measuring room carbon dioxide levels, while not a proxy for infection risks, is a cost-effective tool for identifying poorly ventilated spaces and spaces that have frequent overcrowding, thus indicating places where transmission is likely to occur (58, 72, 73). Poor indoor air quality, measured with carbon dioxide (CO_2_) as a proxy, has been shown to increase lower respiratory tract infections in children (74), more frequent incidences of common cold (75), and even a pneumococcal outbreak in a correctional facility (76).

### Filtration for Indoor Spaces

While introducing outdoor air and increasing ventilation is a preferred option, it also carries energy and hence economic implications. In such a situation, assuming the existing heating ventilation and air conditioning (HVAC) system can handle better grade filters, choosing high-efficiency filters can mitigate risks of infection while requiring less operational costs than increasing the outdoor air ventilation levels (77). The added cost due to improved filtration can far outweigh the cost of infections.

**Figure 1:**
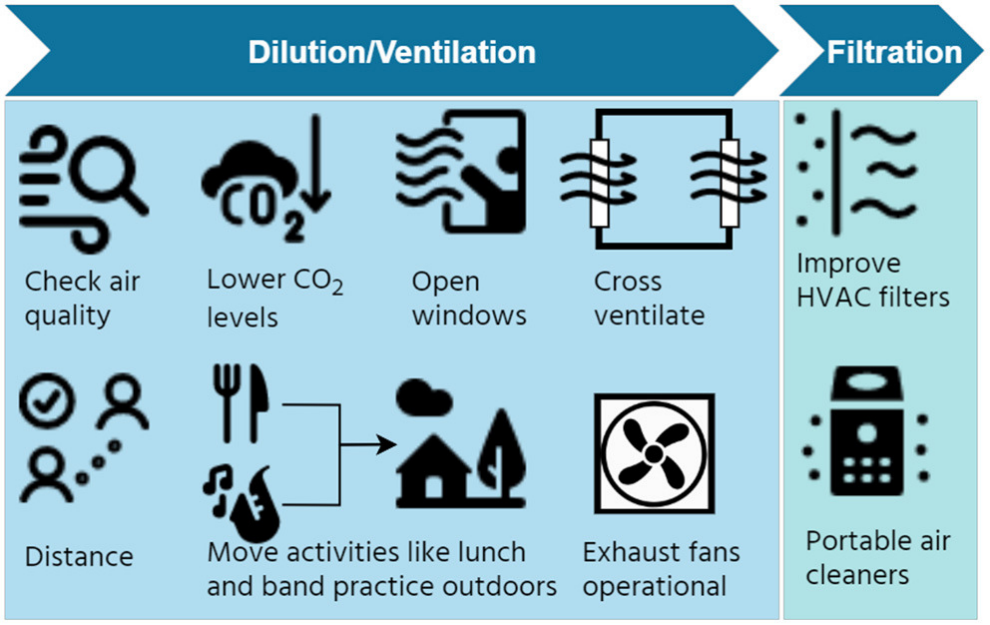
Clean indoor air: dilution, ventilation, and filtration based strategies.

But changing mechanical ventilation in a building can be expensive and time taking. For such situations and also for buildings without mechanical ventilation, use of portable air cleaners (PACs) can be a quick and affordable option. PACs were already in the market since late 70s, early 80s and their use in homes has been increasing, due to a concern with outdoor air quality (78–80). They are part of design recommendations for setting up temporary, negative pressure isolation units (81, 82) and also a part of the WHO Roadmap for ventilation in face of the COVID-19 pandemic (57). Multiple studies during the past months have focused on PACs, due to the ongoing pandemic, and have used approaches CFD modeling (83), experiments in actual spaces (84–88), and study involving actual COVID-19 patients (89) to validate that PACs are an effective mitigation measure. The studies using PACs, to date, have mostly focused on particulate matter pollution (80). Recent studies, cited above, looking at infection mitigation potential have certain limitations in terms of use of different kinds of equipment in different sized spaces, introduction of PACs as part of several other mitigation measures, and few studies that can offer clinical evidence (85, 86, 88, 89). This is an aspect that is gradually starting to gain attention with better designed studies and controlled trials in clinical settings. In the coming years, the noted shortcomings regarding effectiveness of PACs are likely to be comprehensively addressed. The schema in Figure 1 summarizes the mitigation strategies we discuss, centred around improving indoor air quality, through dilution, ventilation, and filtration.

## Author Contributions

All authors listed have made a substantial, direct, and intellectual contribution to the work and approved it for publication.

## Conflict of Interest

The authors declare that the research was conducted in the absence of any commercial or financial relationships that could be construed as a potential conflict of interest.

## Publisher's Note

All claims expressed in this article are solely those of the authors and do not necessarily represent those of their affiliated organizations, or those of the publisher, the editors and the reviewers. Any product that may be evaluated in this article, or claim that may be made by its manufacturer, is not guaranteed or endorsed by the publisher.
